# Adverse event profile of lorazepam: a real-world pharmacovigilance study using the FDA adverse event reporting system database

**DOI:** 10.3389/fphar.2024.1465245

**Published:** 2024-11-22

**Authors:** Zhengkang Su, Zhengwei Huang, Xiaoyu Chen, Xi Li

**Affiliations:** ^1^ The Affiliated Kangning Hospital of Wenzhou Medical University, Zhejiang Provincial Clinical Research Center for Mental Health, Wenzhou, Zhejiang, China; ^2^ Department of Hepatobiliary and Pancreatic Surgery, The First Affiliated Hospital of Wenzhou Medical University, Wenzhou, China

**Keywords:** anxiety, lorazepam, FAERS, adverse events, data mining

## Abstract

**Introduction:**

Anxiety diagnoses have surged recently during and after the COVID-19 pandemic. Lorazepam is widely recognized for its efficacy in treatment of anxiety, as well as insomnia, etc. However, the long-term safety profile of lorazepam in extensive patient populations has not been thoroughly established.

**Methods:**

This study aims to evaluate the potential lorazepam-associated adverse events (AEs) using data mining of the Food and Drug Administration Adverse Event Reporting System (FAERS) of the United States, seeking to provide a guidance for the future therapeutic practices.

**Results:**

Our study revealed drug abuse, suicide attempt, sopor, delirium, and psychotic disorder were among the most prevalent AEs linked to lorazepam. In addition to common AEs, we also found that patients using lorazepam may have the risk of abnormal fat metabolism, cardiac impairment, and immunosuppression-related disorders.

**Discussion:**

In general, our research has unveiled novel AE signals and expanded our understanding of the safety profile of lorazepam in clinical practices, providing guidance for its rational use.

## 1 Introduction

Anxiety is a mental condition featured by unease, worry, or fear that can be triggered by a variety of factors, such as stressful life situations, uncertainties regarding the future, or personal vulnerabilities, which may cause serious harm to individual psychological wellbeing, and the overall harmony of society. Anxiety-related symptoms are one of major mental health disorders, with a weighted prevalence of about 20% in China (Tang et al., 2024). With the intensification of social competition, anxiety seems to have become a common aspect of daily life for many individuals (Nekar et al., 2023; Rabby et al., 2023). Especially during and after the COVID-19 pandemic, the prevalence of anxiety increased in children, adolescents, and young adults (Scott et al., 2023; Fischer-Grote et al., 2024).

The etiologies of anxiety are complex and multifaceted, encompassing genetic predispositions, and psychological factors and environmental influences. Consequently, the treatment strategies of anxiety are also diversified, usually involving in a combination of psychological therapies and pharmacological interventions (Bandelow et al., 2023; Goldstein, 2024). Lorazepam, a benzodiazepine (BDZ) medication, exerts sedative and anti-anxiety effects by enhancing inhibitory circuit in the hippocampus and inhibiting excitability in the central nervous system (CNS). Lorazepam is a prominent and effective psychotropic drug approved by the Food and Drug Administration (FDA) in the treatment of anxiety in clinical practices, as well as in the treatment of other conditions such as insomnia (Ameer and Greenblatt, 1981). As lorazepam becomes more widely used, a range of adverse effects have been identified, predominantly impacting the central nervous system (such as drowsiness, dizziness), mental health (such as emotional fluctuations, fantasies), and causing withdrawal symptoms (Neale and Smith, 2007; Cosci and Chouinard, 2020; Pham Nguyen et al., 2022).

The FDA Adverse Event Reporting System (FAERS) is a database designed for monitoring the post-market drugs and therapeutic bioproducts, which covers tens of millions of case reports of AEs submitted by physicians, pharmacists, manufacturers, healthcare professionals, and others. This database includes all AEs information and medication error information collected by the FDA and serves as a critical approach for evaluating drug safety (Yu et al., 2024; Zhou et al., 2024).

In this study, we aimed to delve into the data concerning lorazepam from the FAERS database over the last 20 years, to retrospectively summarize the AEs of lorazepam, dig out potential unreported AEs and provide guidance for its rational use.

## 2 Methods

### 2.1 Data source

This study collected the American Standard Code for Information Interchange (ASCII) report files from the FAERS database for the period from the first quarter of 2004 to the fourth quarter of 2023 (2004Q1 - 2023Q4). The FAERS data file includes patient demographic and administrative information (DEMO), drug information (DRUG), reaction (REAC), patient outcomes (OUTC), report sources (RPSR), therapy start dates and end dates for reported drugs (THER), and indications for drug administration (INDI). The data was imported into MySQL 15.0 and processed using Navicat Premium 15 software.

### 2.2 Signal filtering and categorization

Drug names were standardized using Medex_UIMA_1.8.3 system. All the AEs documented in the FAERS database were coded by Medical Dictionary for Regulatory Activities 24.0 (MedDRA). During the initial screening phase, we selected preferred terms (PTs) with a reported frequency ≥3 for further analysis. These PTs, along with their corresponding System Organ Class (SOCs) in MedDRA were employed to systematically categorize and analyze the signals.

### 2.3 Data extraction and analysis

Reports suggesting that lorazepam as the primary drug associated with adverse events (AEs) were extracted. Various signal quantification techniques including the reporting odds ratios (ROR), proportional reporting ratios (PRR), Bayesian confidence propagation neural network (BCPNN), and empirical Bayesian geometric mean (EBGM) from the disproportionality methods were employed to evaluate the data from different perspectives to offer a more comprehensive and reliable outcome and rapidly detect rare and unpredictable adverse drug reactions with strong drug-attributable component (Shu et al., 2022). The ROR is a measure of association that compares the odds of an AE occurring for users of a specific drug to the odds of it occurring for non-users, which is calculated by comparing the number of reports for the drug and AE to the number of reports for other drugs and the adverse event. The PRR is a frequency-based method that compares the reporting rate of an AE for a specific drug to the reporting rate for all drugs, which measures how often an AE is reported for a drug relative to all other drugs. BCPNN is used for signal detection in pharmacovigilance, analyzing AE reports to identify potential safety signals associated with medications, and EBGM is a Bayesian method used to assess the strength of the association between a specific drug and an adverse event (AE), which adjusts for the overall reporting rate of the AE across all drugs and provides a measure that is less sensitive to random variation.

The detailed formula for the above methods and prerequisites (Table 1) were listed in below.

**TABLE 1:** 2 × 2 fourfold table of disproportionality method.

	Target AEs	Non-target AEs
Lorazepam	a	b
Non-Lorazepam	c	d

#### 2.3.1 ROR formula

$$ROR=\frac{\text{ad}}{\text{bc}}$$

$$95\%CI=e^{\ln\left(ROR\right)\pm 1.96\sqrt{\frac{1}{a}+\frac{1}{b}+\frac{1}{c}+\frac{1}{d}}}$$

The threshold of positive AE signals: reported cases ≥3 and 95% CI (lower limit) > 1, suggesting that use of the drug may be associated with an increased risk of the AE;

#### 2.3.2 PRR formula

$$PPR=\frac{a\left(c+d\right)}{c\left(a+b\right)}$$

$$\chi^{2}=\frac{{\left(\text{ad}-\text{bc}\right)}^{2}\left(a+b+c+d\right)}{\left(a+b\right)\left(c+d\right)\left(a+c\right)\left(b+d\right)}$$

The threshold of positive AE signals: PRR ≥2, $\chi^{2}$ ≥ 4, reported cases ≥3, and *p* < 0.05, indicating that the AE is reported more frequently for the specific drug than would be expected by chance;

#### 2.3.3 BCPNN formula

$${IC=\log}_{2}\frac{a\left(a+b+c+d\right)}{\left(a+b\right)\left(a+c\right)}$$

$$95\%\text{CI}=E\left(\text{IC}\right)\pm 2\times \sqrt{V\left(\text{IC}\right)}$$

The threshold of positive AE signals: IC025 (the lower limit of 95% CI) > 0, suggesting a possible association between the drug and the adverse event;

#### 2.3.4 EBGM formula

$$EBGM=\frac{\text{aN}}{\left(a+c\right)\left(a+b\right)}$$

$$95\%CI=e^{\ln\left(EBGM\right)\pm 1.96\sqrt{\frac{1}{a}+\frac{1}{b}+\frac{1}{c}+\frac{1}{d}}}$$

The threshold of positive AE signals: EBGM05 (the lower limit of 95% CI) > 2, suggesting a possible association between the drug and the adverse event.

## 3 Results

### 3.1 Descriptive results

**FIGURE 1:**
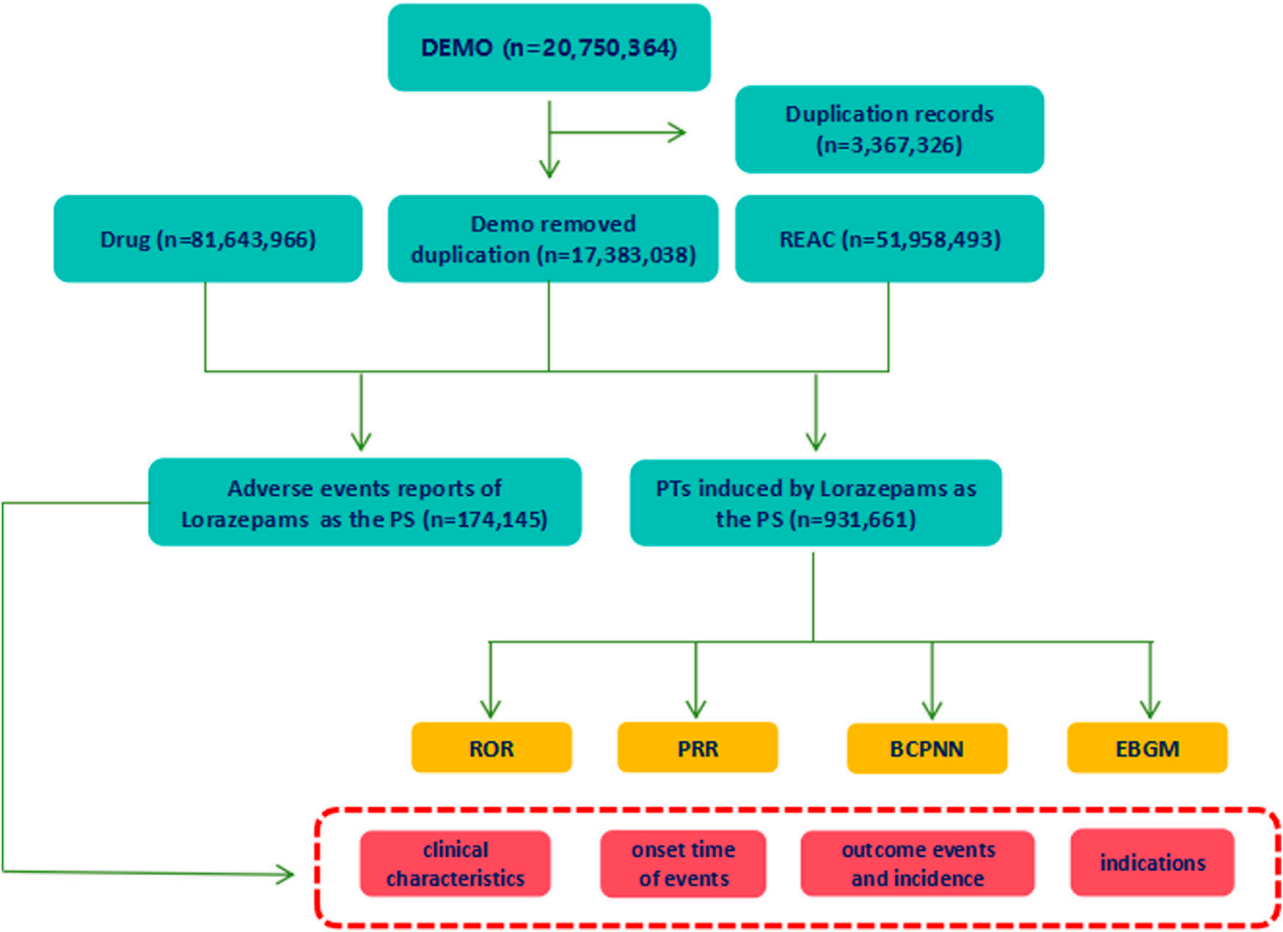
The process of selecting lorazepam-associated adverse events (AEs) from Food and Drug Administration adverse event reporting database (FAERS). Duplicate reports were removed according to the CASEID and FDA_DT. Out of total reports of 17, 383, 038, 174,145 AEs reports that lorazepam was believed as the primarily suspected (PS) drug that causes AEs, and 931,661 preferred terms (PTs) induced by lorazepam (as the primarily suspected drug) were extracted. The basic information of lorazepam-associated reports was collected. And the signal strengths of AEs at PTs levels were detected using ROR, PRR, BCPNN, EBGM methods.

**FIGURE 2:**
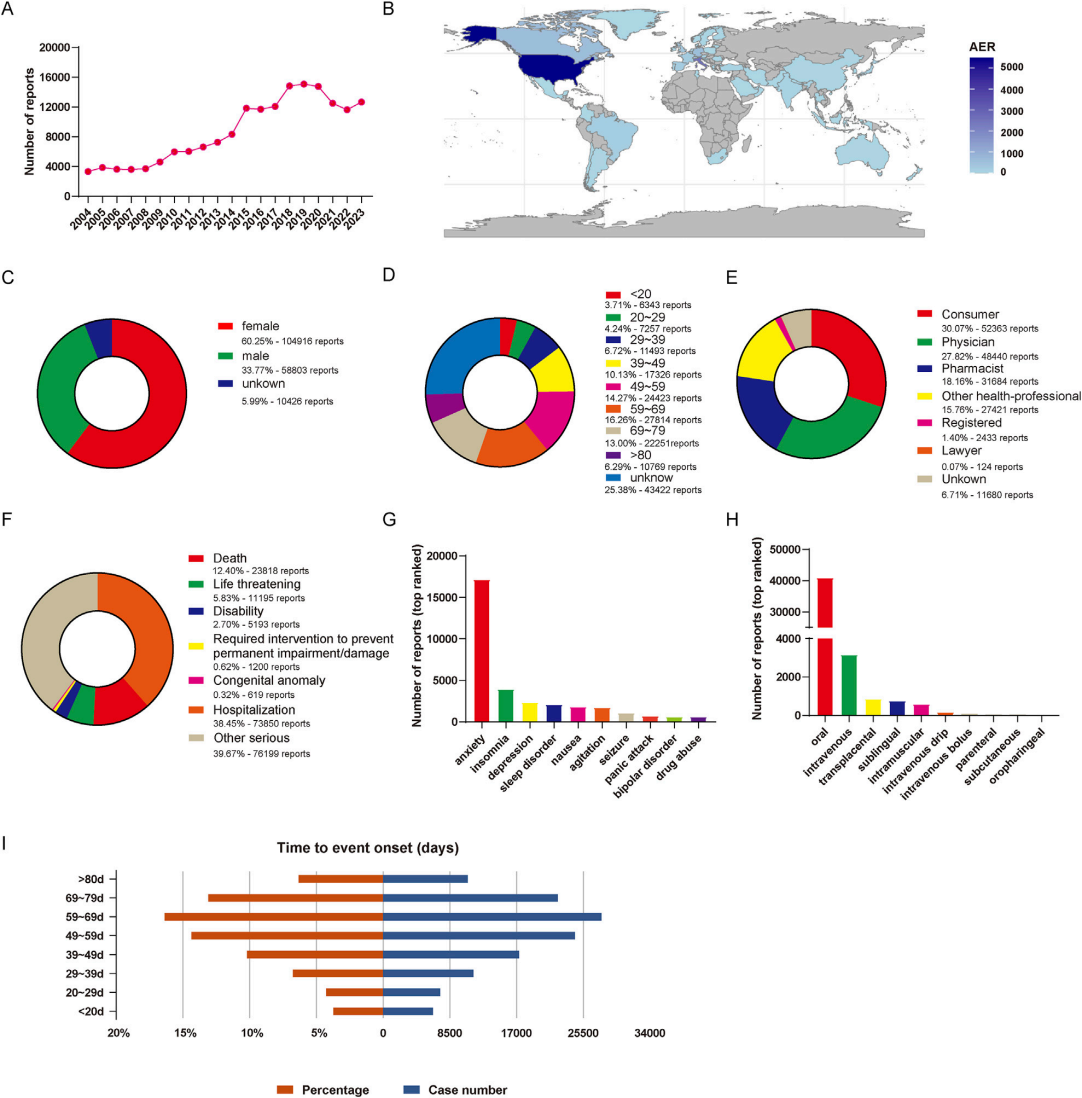
Basic information of lorazepam-associated AE reports in the FAERS from 2004 Q1 to 2023 Q4. **(A)** The annual number of AE reports. **(B)** The world map of countries that report lorazepam-associated AEs. **(C)** The gender distribution of patients. **(D)** The age distribution of patients. **(E)** The occupational distribution of the reporter. **(F)** The outcome distribution of AE reports in patients. **(G)** Top 10 ranked indications distribution of lorazepam. **(H)** Top 10 ranked routes distribution of lorazepam. **(I)** Distribution of the time of AEs occurrence.

From January 2004 to September 2023, a total of 20,750,364 reports were obtained from the FAERS database. Following the FDA’s guidelines for identifying duplicates using CASEID and FDA_DT, we removed the redundant entries. Consequently, after the exclusion of 3,367,326 duplicate reports, a final dataset comprising 17, 383, 038 reports was obtained (Figure 1). There were 174,145 reports of lorazepam as the primarily suspected (PS) drug, and 931,661 AEs preferred terms (PTs) induced by lorazepam (as the primarily suspected drug) were identified. The detailed information of lorazepam-associated adverse events reports was described in Figure 2. Since 2004, the number of AE reports of lorazepam has gradually increased, with the highest volume recorded in 2019 (15,079 reports). The majority of these AE reports came from United States (59.79%), followed by Canada (10.55%), Italy (5.38%), Germany (3.73%), and United Kingdom (3.26%). The AE reports were mainly submitted by consumers (30.07%), physician (27.82%), pharmacist (18.19%) and other health-professional (15.75%), which were consistent with indications approved by FDA. Among all reports, females (60.25%) accounted for a larger proportion than males (33.77%). Patients were mainly aged >20 years old in the reports recording age (95.03%). Hospitalization (38.45%) was the most frequently reported serious outcome. Additionally, death or life-threatening events were reported in 23,818 cases (12.40%) and 11,195 cases (5.83%), respectively. The administration of lorazepam was predominantly oral, with 87.48% of the reports indicating this route of administration. Notably, a significant percentage of AEs occurred within the first 80 days of treatment, representing 91.57% of the cases.

### 3.2 AE signal mining results

We firstly investigated the signal strength of lorazepam-associated AEs at the System Organ Class (SOC) level (Table 2). Statistically, we found that lorazepam-induced AEs occurrence targeted 24 SOCs. The top three AEs occurred and ranked by case numbers were general disorders (symptoms like fatigue, fever, chills, edema (swelling), and other general systemic effects) and administration site conditions (local adverse effects, such as inflammation, pain, or infection, that manifest at the location where a drug is applied or injected) (126,277 cases, ROR (95% CI) = 0.74 (0.74, 0.74), PRR (95% CI) = 0.78 (0.78, 0.78), IC (IC025) = −0.36 (−0.37), EBGM (EBGM05) = 0.78 (0.78)), nervous system disorders (97,378 cases, ROR (95% CI) = 1.25 (1.25, 1.26), PRR (95% CI) = 1.23 (1.23, 1.23), IC (IC025) = 0.29 (0.28), EBGM (EBGM05) = 1.22 (1.22)), psychiatric disorders (97,378 cases, ROR (95% CI) = 1.89 (1.88, 1.9), PRR (95% CI) = 1.79 (1.79, 1.79), IC (IC025) = 0.82 (0.81), EBGM (EBGM05) = 1.77 (1.76)), which is consistent with the common AEs of lorazepam. The significant SOCs that at least one of the four algorithm meets the criteria were psychiatric disorders (IC (IC025) = 0.82 (0.81)), metabolism and nutrition disorders (IC (IC025) = 0.46 (0.45)), nervous system disorders, investigations (IC (IC025) = 0.29 (0.28)), investigations (AE that related to clinical laboratory tests and other diagnostic procedures) (IC (IC025) = 0.25 (0.24)), blood and lymphatic system disorders (IC (IC025) = 0.23 (0.21)), endocrine disorders (IC (IC025) = 0.21 (0.15)), renal and urinary disorders (IC (IC025) = 0.18 (0.16)), cardiac disorders (IC (IC025) = 0.18 (0.16)), respiratory, thoracic and mediastinal disorders (IC (IC025) = 0.13 (0.12)), gastrointestinal disorders (IC (IC025) = 0.09 (0.08)), vascular disorders (IC (IC025) = 0.04 (0.02)), musculoskeletal and connective tissue disorders (IC (IC025) = 0.02 (0.01)).

**TABLE 2:** Signal strength of lorazepam-associated AEs at the System Organ Class (SOC) level in the FAERS database.

System organ class	Preferred terms	ROR (95% CI)	PRR (95% CI)	IC(IC025)	EBGM(EBGM05)
Psychiatric disorders	95,414	1.89 (1.88, 1.9)	1.79 (1.79, 1.79)	0.82 (0.81)	1.77 (1.76)
Metabolism and nutrition disorders	27,688	1.4 (1.38, 1.42)	1.39 (1.36, 1.42)	0.46 (0.45)	1.38 (1.37)
Nervous system disorders	97,378	1.25 (1.25, 1.26)	1.23 (1.23, 1.23)	0.29 (0.28)	1.22 (1.22)
Investigations	69,184	1.21 (1.2, 1.22)	1.2 (1.2, 1.2)	0.25 (0.24)	1.19 (1.18)
Blood and lymphatic system disorders	18,567	1.18 (1.17, 1.2)	1.18 (1.16, 1.2)	0.23 (0.21)	1.18 (1.16)
Endocrine disorders	2,703	1.16 (1.12, 1.2)	1.16 (1.12, 1.21)	0.21 (0.15)	1.16 (1.12)
Renal and urinary disorders	19,698	1.14 (1.13, 1.16)	1.14 (1.12, 1.16)	0.18 (0.16)	1.14 (1.12)
Cardiac disorders	28,642	1.14 (1.13, 1.16)	1.14 (1.12, 1.16)	0.18 (0.16)	1.13 (1.12)
Respiratory, thoracic and mediastinal disorders	48,705	1.1 (1.09, 1.11)	1.1 (1.1, 1.1)	0.13 (0.12)	1.1 (1.09)
Gastrointestinal disorders	84,913	1.07 (1.07, 1.08)	1.07 (1.07, 1.07)	0.09 (0.08)	1.06 (1.06)
Vascular disorders	20,943	1.03 (1.02, 1.04)	1.03 (1.01, 1.05)	0.04 (0.02)	1.03 (1.02)
Musculoskeletal and connective tissue disorders	50,256	1.02 (1.01, 1.03)	1.02 (1.02, 1.02)	0.02 (0.01)	1.02 (1.01)
Hepatobiliary disorders	8,165	0.96 (0.94, 0.98)	0.96 (0.94, 0.98)	−0.06 (-0.09)	0.96 (0.94)
Infections and infestations	46,518	0.95 (0.94, 0.96)	0.96 (0.96, 0.96)	−0.06 (-0.08)	0.96 (0.95)
Ear and labyrinth disorders	3,661	0.91 (0.88, 0.94)	0.91 (0.88, 0.95)	−0.14 (-0.19)	0.91 (0.88)
Injury, poisoning and procedural complications	66,754	0.78 (0.77, 0.78)	0.79 (0.79, 0.79)	−0.33 (-0.34)	0.8 (0.79)
General disorders and administration site conditions	126,277	0.74 (0.74, 0.74)	0.78 (0.78, 0.78)	−0.36 (-0.37)	0.78 (0.78)
Congenital, familial and genetic disorders	2020	0.69 (0.66, 0.72)	0.69 (0.66, 0.72)	−0.53 (-0.59)	0.69 (0.67)
Eye disorders	12,891	0.69 (0.67, 0.7)	0.69 (0.68, 0.7)	−0.53 (-0.55)	0.69 (0.68)
Skin and subcutaneous tissue disorders	34,941	0.69 (0.68, 0.69)	0.7 (0.69, 0.71)	−0.51 (-0.53)	0.7 (0.7)
Immune system disorders	7,102	0.69 (0.67, 0.7)	0.69 (0.68, 0.7)	−0.53 (-0.57)	0.69 (0.68)
Reproductive system and breast disorders	4,432	0.56 (0.54, 0.58)	0.56 (0.54, 0.58)	−0.82 (-0.86)	0.57 (0.55)
Neoplasms benign, malignant and unspecified (incl cysts and polyps)	13,252	0.51 (0.5, 0.52)	0.52 (0.51, 0.53)	−0.93 (-0.95)	0.53 (0.52)
Pregnancy, puerperium and perinatal conditions	1884	0.46 (0.44, 0.48)	0.46 (0.44, 0.48)	−1.11 (-1.18)	0.46 (0.45)

ROR, reporting odds ratio; PRR, proportional reporting ratio; IC, information component; EBGM, empirical bayes geometric mean.

We next investigated the most frequent lorazepam-associated AEs at preferred terms (PT) level. A total of 552 signals of lorazepam-induced AEs were detected after conforming to the four algorithms simultaneously. And the number of case reporting AEs >300 was presented in Table 3, including 33 PTs and 13 corresponding SOCs. Notably, drug abuse (4,135 cases, ROR (95% CI) = 3.29 (3.19, 3.4), PRR (95% CI) = 3.28 (3.15, 3.41), IC (IC025) = 1.66 (1.61), EBGM (EBGM05) = 3.15 (3.07)), suicide attempt (2,866 cases, ROR (95% CI) = 3.13 (3.01, 3.25), PRR (95% CI) = 3.12 (3, 3.24), IC (IC025) = 1.59 (1.53), EBGM (EBGM05) = 1.59 (1.53)), sopor (2,407 cases, ROR (95% CI) = 13.1 (12.53, 13.69), PRR (95% CI) = 13.06 (12.56, 13.58), IC (IC025) = 3.42 (3.36), EBGM (EBGM05) = 10.7 (10.31)), delirium (1896 cases, ROR (95% CI) = 3.84 (3.67, 4.03), PRR (95% CI) = 3.84 (3.69, 3.99), IC (IC025) = 1.87 (1.8), EBGM (EBGM05) = 3.65 (3.51)), psychotic disorder (1,441 cases, ROR (95% CI) = 3.21 (3.05, 3.39), PRR (95% CI) = 3.21 (3.03, 3.4), IC (IC025) = 1.63 (1.55), EBGM (EBGM05) = 3.09 (2.95)) had high signal frequencies, aligning with the drug’s label information of lorazepam. Of note, a lot of unexpected significant AEs that uncovered in the label were found in our data mining, such as PTs of pneumonia aspiration (1,178 cases, ROR (95% CI) = 3.19 (3.01, 3.39), PRR (95% CI) = 3.19 (3.01, 3.38), IC (IC025) = 1.62 (1.53), EBGM (EBGM05) = 3.07 (2.92)), obesity (709 cases, ROR (95% CI) = 3.07 (2.92), PRR (95% CI) = 3.18 (2.94, 3.44), IC (IC025) = 1.61 (1.5), EBGM (EBGM05) = 3.06 (2.87)), sinus tachycardia (676 cases, ROR (95% CI) = 3.11 (2.88, 3.36), PRR (95% CI) = 3.11 (2.88, 3.36), IC (IC025) = 1.58 (1.47), EBGM (EBGM05) = 2.99 (2.8)), pericarditis (629 cases, ROR (95% CI) = 3.09 (2.85, 3.35), PRR (95% CI) = 3.09 (2.85, 3.35), IC (IC025) = 1.57 (1.46), EBGM (EBGM05) = 2.97 (2.78)), duodenal ulcer perforation (507 cases, ROR (95% CI) = 7.99 (7.28, 8.77), PRR (95% CI) = 7.99 (7.24, 8.81), IC (IC025) = 2.82 (2.69), EBGM (EBGM05) = 7.08 (6.55)).

**TABLE 3:** The top 30 signal strength of lorazepam-associated AEs ranked by AE numbers at the PTs level in FAERS database.

System organ class	Preferred terms	Reported Cases	ROR (95% CI)	PRR (95% CI)	IC(IC025)	EBGM(EBGM05)
Metabolism and nutrition disorders	hypercholesterolaemia	374	3 (2.71, 3.33)	3 (2.72, 3.31)	1.53 (1.38)	2.9 (2.65)
Metabolism and nutrition disorders	obesity	709	3.18 (2.95, 3.43)	3.18 (2.94, 3.44)	1.61 (1.5)	3.06 (2.87)
Infections and infestations	folliculitis	497	4.69 (4.28, 5.14)	4.69 (4.25, 5.17)	2.13 (2)	4.39 (4.07)
Infections and infestations	pneumonia aspiration	1,178	3.19 (3.01, 3.39)	3.19 (3.01, 3.38)	1.62 (1.53)	3.07 (2.92)
Investigations	c-reactive protein abnormal	320	4.46 (3.98, 5)	4.46 (3.97, 5.02)	2.07 (1.9)	4.19 (3.81)
Investigations	anti-cyclic citrullinated peptide antibody positive	373	5.23 (4.71, 5.82)	5.23 (4.74, 5.77)	2.28 (2.13)	4.86 (4.44)
Nervous system disorders	bradykinesia	332	4.38 (3.92, 4.9)	4.38 (3.89, 4.93)	2.04 (1.88)	4.12 (3.75)
Nervous system disorders	parkinsonism	437	3.15 (2.86, 3.47)	3.15 (2.86, 3.47)	1.6 (1.46)	3.03 (2.79)
Nervous system disorders	status epilepticus	556	3.36 (3.08, 3.66)	3.36 (3.11, 3.63)	1.69 (1.56)	3.22 (3)
Nervous system disorders	serotonin syndrome	1,043	3.94 (3.7, 4.2)	3.94 (3.72, 4.18)	1.9 (1.81)	3.74 (3.54)
Nervous system disorders	sedation	1,249	3.47 (3.28, 3.67)	3.46 (3.26, 3.67)	1.73 (1.65)	3.32 (3.16)
Nervous system disorders	neuroleptic malignant syndrome	1,267	7.63 (7.2, 8.09)	7.62 (7.18, 8.08)	2.77 (2.68)	6.8 (6.47)
Injury, poisoning and procedural complications	muscle injury	435	5.01 (4.54, 5.52)	5 (4.53, 5.51)	2.22 (2.08)	4.66 (4.29)
Musculoskeletal and connective tissue disorders	muscle rigidity	600	3.35 (3.09, 3.64)	3.35 (3.1, 3.62)	1.68 (1.57)	3.21 (3)
Respiratory, thoracic and mediastinal disorders	atelectasis	549	3.62 (3.32, 3.95)	3.62 (3.35, 3.92)	1.79 (1.66)	3.45 (3.21)
Respiratory, thoracic and mediastinal disorders	respiratory depression	693	3.73 (3.45, 4.02)	3.72 (3.44, 4.02)	1.83 (1.72)	3.55 (3.33)
Psychiatric disorders	sleep disorder due to general medical condition, insomnia type	308	4.5 (4, 5.05)	4.5 (4, 5.06)	2.08 (1.91)	4.23 (3.83)
Psychiatric disorders	major depression	373	3.45 (3.11, 3.84)	3.45 (3.13, 3.81)	1.72 (1.57)	3.3 (3.03)
Psychiatric disorders	schizophrenia	629	3.61 (3.33, 3.92)	3.61 (3.34, 3.9)	1.79 (1.67)	3.45 (3.22)
Psychiatric disorders	bradyphrenia	641	6.53 (6.02, 7.09)	6.53 (6.04, 7.06)	2.57 (2.45)	5.93 (5.54)
Psychiatric disorders	catatonia	745	11.47 (10.59, 12.41)	11.46 (10.6, 12.39)	3.27 (3.15)	9.62 (9)
Psychiatric disorders	hallucination, auditory	761	3.2 (2.97, 3.44)	3.2 (2.96, 3.46)	1.62 (1.52)	3.07 (2.89)
Psychiatric disorders	intentional self-injury	1,310	4.39 (4.15, 4.65)	4.39 (4.14, 4.66)	2.05 (1.97)	4.13 (3.94)
psychiatric disorders	psychotic disorder	1,441	3.21 (3.05, 3.39)	3.21 (3.03, 3.4)	1.63 (1.55)	3.09 (2.95)
Psychiatric disorders	delirium	1896	3.84 (3.67, 4.03)	3.84 (3.69, 3.99)	1.87 (1.8)	3.65 (3.51)
Psychiatric disorders	sopor	2,407	13.1 (12.53, 13.69)	13.06 (12.56, 13.58)	3.42 (3.36)	10.7 (10.31)
Psychiatric disorders	suicide attempt	2,866	3.13 (3.01, 3.25)	3.12 (3, 3.24)	1.59 (1.53)	3.01 (2.91)
Psychiatric disorders	drug abuse	4,135	3.29 (3.19, 3.4)	3.28 (3.15, 3.41)	1.66 (1.61)	3.15 (3.07)
Skin and subcutaneous tissue disorders	pemphigus	489	3.31 (3.02, 3.63)	3.31 (3, 3.65)	1.67 (1.54)	3.18 (2.94)
Gastrointestinal disorders	duodenal ulcer perforation	507	7.99 (7.28, 8.77)	7.99 (7.24, 8.81)	2.82 (2.69)	7.08 (6.55)
Cardiac disorders	pericarditis	629	3.09 (2.85, 3.35)	3.09 (2.86, 3.34)	1.57 (1.46)	2.97 (2.78)
Cardiac disorders	sinus tachycardia	676	3.11 (2.88, 3.36)	3.11 (2.88, 3.36)	1.58 (1.47)	2.99 (2.8)
Blood and lymphatic system disorders	nephrogenic anaemia	352	4.61 (4.14, 5.14)	4.61 (4.1, 5.19)	2.11 (1.96)	4.32 (3.95)

In our analysis, we have not only focused on AEs with a high volume of reports but have also identified the top 30 AEs with significant signal strength ranked by EBGM, which are listed in Table 4. The top five AEs with strongest signal were natural killer cell count (4 cases, ROR (95% CI) = 214.65 (23.99, 1920.5), PRR (95% CI) = 214.65 (23.9, 1927.94), IC (IC025) = 5.45 (3.74), EBGM (EBGM05) = 43.73 (6.99)), affective ambivalence (4 cases, ROR (95% CI) = 214.65 (23.99, 1920.5), PRR (95% CI) = 214.65 (23.9, 1927.94), IC (IC025) = 5.45 (3.74), EBGM (EBGM05) = 43.73 (6.99)), morbihan disease (4 cases, ROR (95% CI) = 214.65 (23.99, 1920.5), PRR (95% CI) = 214.65 (23.9, 1927.94), IC (IC025) = 5.45 (3.74), EBGM (EBGM05) = 43.73 (6.99)), computerised tomogram thorax (36 cases, ROR (95% CI) = 175.63 (89.4, 345.02), PRR (95% CI) = 175.62 (90.19, 341.97), IC (IC025) = 5.39 (4.77), EBGM (EBGM05) = 41.87 (23.8)), and parachute mitral valve (9 cases, ROR (95% CI) = 160.99 (43.58, 594.66), PRR (95% CI) = 160.98 (43.3, 598.53), IC (IC025) = 5.36 (4.17), EBGM (EBGM05) = 41 (13.74)). Additionally, we have also observed that AEs with strong signals that have been reported with relatively higher frequency, such as deep vein thrombosis postoperative (275 cases, ROR (95% CI) = 42.42 (36.21, 49.69, PRR (95% CI) = 42.4 (36.25, 49.6), IC (IC025) = 4.59 (4.39), EBGM (EBGM05) = 24.13 (21.14)), lupus vulgaris (99 cases, ROR (95% CI) = 33 (25.69, 42.39), PRR (95% CI) = 33 (25.58, 42.58), IC (IC025) = 4.38 (4.05), EBGM (EBGM05) = 20.81 (16.88)). The combination of a strong signal with a higher frequency of reported cases for these AEs suggests a potential increased risk associated with lorazepam use and warrants close clinical attention and further research to understand their implications fully.

**TABLE 4:** The top 30 signal strength of lorazepam-associated AEs ranked by EBGM at the PTs level in FAERS database.

System organ class	Preferred terms	Reported Cases	ROR (95% CI)	PRR (95% CI)	IC(IC025)	EBGM(EBGM05)
Infections and infestations	lupus vulgaris	99	33 (25.69, 42.39)	33 (25.58, 42.58)	4.38 (4.05)	20.81 (16.88)
Infections and infestations	gastroenteritis *listeria*	4	30.66 (8.98, 104.75)	30.66 (8.92, 105.4)	4.31 (2.81)	19.88 (7.11)
Investigations	natural killer cell count	4	214.65 (23.99, 1920.5)	214.65 (23.9, 1927.94)	5.45 (3.74)	43.73 (6.99)
Investigations	computerised tomogram thorax	36	175.63 (89.4, 345.02)	175.62 (90.19, 341.97)	5.39 (4.77)	41.87 (23.8)
Investigations	barium enema abnormal	3	80.49 (13.45, 481.73)	80.49 (13.52, 479.02)	5.04 (3.21)	32.8 (7.34)
Investigations	maximal voluntary ventilation abnormal	5	53.66 (15.53, 185.36)	53.66 (15.61, 184.47)	4.77 (3.34)	27.33 (9.69)
Investigations	anti-neuronal antibody positive	3	53.66 (10.83, 265.88)	53.66 (10.76, 267.7)	4.77 (3)	27.33 (7.16)
Investigations	forced expiratory volume	16	47.7 (24.32, 93.54)	47.7 (24.5, 92.88)	4.68 (3.85)	25.72 (14.64)
Investigations	dopamine transporter scintigraphy abnormal	9	43.91 (18.19, 105.95)	43.9 (18.17, 106.05)	4.62 (3.53)	24.6 (11.77)
Nervous system disorders	muscle tension dysphonia	20	34.62 (19.73, 60.74)	34.62 (19.61, 61.12)	4.42 (3.69)	21.44 (13.39)
Nervous system disorders	presenile dementia	9	32.2 (14.09, 73.57)	32.2 (14.14, 73.34)	4.36 (3.3)	20.5 (10.27)
Injury, poisoning and procedural complications	deep vein thrombosis postoperative	275	42.42 (36.21, 49.69)	42.4 (36.25, 49.6)	4.59 (4.39)	24.13 (21.14)
Injury, poisoning and procedural complications	post-thoracotomy pain syndrome	4	30.66 (8.98, 104.75)	30.66 (8.92, 105.4)	4.31 (2.81)	19.88 (7.11)
Musculoskeletal and connective tissue disorders	vertebral end plate inflammation	10	134.16 (42.07, 427.75)	134.15 (42.21, 426.39)	5.29 (4.16)	39.04 (14.8)
Musculoskeletal and connective tissue disorders	dysponesis	5	33.54 (10.97, 102.52)	33.54 (10.97, 102.51)	4.39 (3.01)	21.02 (8.25)
Congenital, familial and genetic disorders	parachute mitral valve	9	160.99 (43.58, 594.66)	160.98 (43.3, 598.53)	5.36 (4.17)	41 (13.74)
Congenital, familial and genetic disorders	cryptophthalmos	3	80.49 (13.45, 481.73)	80.49 (13.52, 479.02)	5.04 (3.21)	32.8 (7.34)
Congenital, familial and genetic disorders	trisomy 16	3	32.2 (7.69, 134.73)	32.2 (7.7, 134.66)	4.36 (2.66)	20.5 (6.19)
Congenital, familial and genetic disorders	amegakaryocytic thrombocytopenia	4	30.66 (8.98, 104.75)	30.66 (8.92, 105.4)	4.31 (2.81)	19.88 (7.11)
Psychiatric disorders	affective ambivalence	4	214.65 (23.99, 1920.5)	214.65 (23.9, 1927.94)	5.45 (3.74)	43.73 (6.99)
Psychiatric disorders	clang associations	7	93.91 (27.49, 320.8)	93.91 (27.32, 322.83)	5.12 (3.83)	34.78 (12.44)
Psychiatric disorders	neologism	9	37.15 (15.88, 86.91)	37.15 (15.99, 86.29)	4.48 (3.41)	22.36 (10.98)
Psychiatric disorders	phagophobia	13	33.22 (16.63, 66.34)	33.22 (16.73, 65.97)	4.39 (3.49)	20.9 (11.72)
Neoplasms benign, malignant and unspecified (incl cysts and polyps)	vulvar basal cell carcinoma	5	134.15 (26.03, 691.49)	134.15 (25.86, 696.01)	5.29 (3.76)	39.04 (9.9)
Neoplasms benign, malignant and unspecified (incl cysts and polyps)	malignant cranial nerve neoplasm	18	120.74 (52.5, 277.68)	120.74 (53.01, 275.02)	5.24 (4.4)	37.84 (18.85)
Neoplasms benign, malignant and unspecified (incl cysts and polyps)	retro-orbital neoplasm	19	31.86 (18.06, 56.21)	31.86 (18.05, 56.25)	4.35 (3.6)	20.36 (12.66)
Skin and subcutaneous tissue disorders	morbihan disease	4	214.65 (23.99, 1920.5)	214.65 (23.9, 1927.94)	5.45 (3.74)	43.73 (6.99)
Reproductive system and breast disorders	varicose veins vulval	5	53.66 (15.53, 185.36)	53.66 (15.61, 184.47)	4.77 (3.34)	27.33 (9.69)
Gastrointestinal disorders	pancreatic fibrosis	16	37.33 (19.72, 70.66)	37.33 (19.55, 71.28)	4.49 (3.67)	22.43 (13.15)
Cardiac disorders	myocardial reperfusion injury	17	53.66 (27.4, 105.11)	53.66 (27.56, 104.49)	4.77 (3.95)	27.33 (15.57)

## 4 Discussion

In this study, we carried out a comprehensive and systematic pharmacovigilance data mining on lorazepam-associated AEs reports based on the FAERS database. We strictly collected and analyzed lorazepam-induced AEs over the past 20 years. The reports of lorazepam AEs since 2004 have continued to increase and reach to its peak in 2019 with 15,079 cases, due to the excellent therapeutic effect of the medication, as well as increasing number of patients. In this study, lorazepam showed a high proportion of AEs in older patients with the highest percentage observed in the 59–69 years age group, accounting for 16.26% of the total AEs This demographic trend may be linked to the natural decline in physical functioning and the cumulative effects of social stress experienced by this age group. The proportion of lorazepam utilization in females is nearly twice as high as that in males, which is also related to the fact that females are physically and psychologically more prone to anxiety (Warner et al., 2023). In addition, the outcomes of lorazepam treatment can be less than favorable, with a notable percentage of cases resulting in death (12.4%), life-threatening conditions (5.83%), and other serious consequences (39.67%), there is a pressing need to closely monitor and understand the AEs associated with its use.

As a medication primarily used in the treatment of psychiatric conditions, the most reported and significant SOCs of lorazepam are psychiatric disorders, followed by a range of other SOCs. It is noteworthy that metabolism and nutrition disorders, endocrine disorders, thoracic and mediastinal disorders are not mentioned in the drug’s leaflet. This oversight raises concerns and highlights the need for further research and attention to these potential risks associated with lorazepam use. Among the SOC of psychiatric disorders, most frequently reported in association with lorazepam use include drug abuse, suicide attempt, sopor, delirium, psychotic disorder, intentional self-injury, which have been wildly recognized and documented by numerous studies, highlighting their significance in the safety profile of lorazepam. Additionally, we also observed other common PTs, such as neuroleptic malignant syndrome, sedation (Babakhanian et al., 2012; Friedrich et al., 2020; Gibbons et al., 2024; Midkiff et al., 2024). Notably, we found some unexpected PTs, such as pneumonia aspiration, obesity, sinus tachycardia, pericarditis, duodenal ulcer perforation, pemphigus, hypercholesterolaemia. Pneumonia aspiration, a type of pulmonary infection, is caused by inhalation of oral secretions, gastric contents, or both. This situation may occur in patients with reduced consciousness or swallowing dysfunction stem from various reasons, such as unconscious due to anesthesia, sedatives, or medical conditions (Kong et al., 2022). Lorazepam has the effects of sedation and hypnosis, which may have the potential to cause pneumonia aspiration. Drug induced obesity may occur after taking various medications, such as antipsychotics (clozapine, olanzapine) (Larsen et al., 2017; Miyakoshi et al., 2023), antidepressants (selective serotonin reuptake inhibitors SSRIs) (Suchacki et al., 2023), corticosteroids (glucocorticoids) (Zhang et al., 2024). It was noteworthy that patients used lorazepam may exhibit metabolism and nutrition disorders (obesity and hypercholesterolaemia), suggesting that the underlying influence of lorazepam in fat metabolism. Meanwhile, numerous AE signal of sinus tachycardia and pericarditis were also found, indicating the cardiac impairment potential of lorazepam. The long-term use of non-steroidal anti-inflammatory drugs (NSAIDs), such as aspirin, may damage gastrointestinal mucosa and increase the risk of duodenal ulcer perforation (Niv et al., 2005). At present, there is no report that lorazepam can increase the risk of duodenal ulcer perforation, which may be caused by indirectly affecting gastrointestinal function through affecting the central nervous system. Pemphigus is a group of serious autoimmune bullous diseases characterized by the formation of loose blisters on the skin and mucosa (Timoteo et al., 2024). The occurrence of pemphigus indicated the inhibitory effect of lorazepam in immune system.

Other than common AEs, we also listed some AEs with relatively less occurrence but higher significance (ranked by EBMG). It is obvious that the use of lorazepam is closely related to the occurrence of deep vein thrombosis postoperative. As we mentioned above, lorazepam has good sedative and hypnotic effects, so it is also widely used to relieve postoperative anxiety and pain and promote sleep (Morgan and Malison, 2008). Long-term bed rest after surgery may reduce and slow the blood flow reflux of patients, which leads to deep vein thrombosis, which may explain the occurrence of deep vein thrombosis postoperative in patients used lorazepam. Meanwhile, we also observed the occurrence of lupus vulgaris, a disease caused by the infection of *mycobacterium tuberculosis* (Couppoussamy et al., 2024), which is also an immune related disease, further suggesting the potential of immune inhibition of lorazepam.

It must be emphasized that the above discussion on AEs and their potential mechanism with lorazepam is only preliminary conjecture. Therefore, we must combine clinical and basic research to reach a positive conclusion, when interpreting AEs. At the same time, medical professionals should continue to monitor the occurrence of serious AEs in clinical practice and take intervention measures as soon as possible. Although this study provides a reliable scientific basis for the safety evaluation of lorazepam from multiple perspectives, there are still some limitations. Firstly, the database collects AE reports through a voluntary reporting system, which lacks rigorous oversight of patient confidentiality and actual medication usage. This could result in underreporting, incorrect reporting, or the exclusion of certain data. Meanwhile, our statistical findings merely suggest a link between lorazepam and certain AEs, not a definitive causal relationship. Additionally, there are many confounders that can bias statistical analysis outcomes such as individual health status, gender differences, patients’ existing diseases and unknown concomitant medication use. To obtain a more comprehensive and accurate research perspective, future studies must consider using more rigorous prospective studies combined with clinical trials and epidemiological studies.

## 5 Conclusion

Our pharmacovigilance study explored reports of lorazepam-associated AEs using FAERS database. 174,145 reports of lorazepam as the PS and 931,661 AEs induced by lorazepam were identified over the last 20 years. Common AEs in SOC levels, such as drug abuse, suicide attempt, sopor, delirium, psychotic disorder should be overly concerned. Unexpected and novel significant AEs such as pneumonia aspiration, sinus tachycardia, pericarditis, obesity, hypercholesterolaemia, duodenal ulcer perforation, lupus vulgaris and deep vein thrombosis postoperative and others might also occur. In addition to the common adverse reactions, we found that use of lorazepam may have the potential to lead to abnormal fat metabolism, cardiac impairment, and immunosuppression-related disorders, but this needs further clinical and basic research evidence to prove. In general, our research has identified novel AE signals and expanded our understanding of the safety of lorazepam, providing guidance for its rational use.

## Data Availability

The original contributions presented in the study are included in the article/supplementary material, further inquiries can be directed to the corresponding author.
